# Composite Behavior of Insulated Concrete Sandwich Wall Panels Subjected to Wind Pressure and Suction

**DOI:** 10.3390/ma8031264

**Published:** 2015-03-19

**Authors:** Insub Choi, JunHee Kim, Ho-Ryong Kim

**Affiliations:** 1 Department of Architectural Engineering, Yonsei University, 50 Yonseiro, Seodaemun-gu, Seoul 120-749, Korea; E-Mail: insub@yonsei.ac.kr; 2 Building Research Department, Korea Institute of Civil Engineering and Building Technology, 283 Goyangdae-ro, Gogang-si, Gyeonggi-do 411-712, Korea; E-Mail: horyong83@nate.com

**Keywords:** composite behavior, wind pressure, wind suction, GFRP continuous shear connector, flexural strength, bond strength, insulated concrete sandwich wall panels

## Abstract

A full-scale experimental test was conducted to analyze the composite behavior of insulated concrete sandwich wall panels (ICSWPs) subjected to wind pressure and suction. The experimental program was composed of three groups of ICSWP specimens, each with a different type of insulation and number of glass-fiber-reinforced polymer (GFRP) shear grids. The degree of composite action of each specimen was analyzed according to the load direction, type of the insulation, and number of GFRP shear grids by comparing the theoretical and experimental values. The failure modes of the ICSWPs were compared to investigate the effect of bonds according to the load direction and type of insulation. Bonds based on insulation absorptiveness were effective to result in the composite behavior of ICSWP under positive loading tests only, while bonds based on insulation surface roughness were effective under both positive and negative loading tests. Therefore, the composite behavior based on surface roughness can be applied to the calculation of the design strength of ICSWPs with continuous GFRP shear connectors.

## 1. Introduction

Insulation is a basic aspect of passive construction for reducing energy through eco-friendly and highly-efficient air-conditioning and heating systems; together with new and renewable energies, insulation is the most important element in zero-energy construction. While external insulation methods may offer energy-saving effects up to the level of zero energy and zero carbon, complex construction and durability issues are impediments to full realization in the field. The insulated concrete sandwich wall panel (ICSWP), which consists of an insulating material and internal/external concrete wythes, is a good alternative that can satisfy the levels of both structural and insulation performance required for buildings. For ICSWP to be used as an efficient load-bearing element, however, it is necessary to improve its structural performance by increasing the degree of composite of the internal/external concrete wythes.

The degree of composite action can be classified into full-composite, partial-composite, and non-composite, and is determined by the shear flow capacity based on the shear connector and bond strength between the insulation and concrete panels. The capacity of the shear connector is determined by material and geometry, and is more effective for the application of continuous shear [1]. While the solid concrete rib shows full-composite action [2,3], its thermal performance is poor. Several studies conducted the test using steel, fiber-reinforced polymer (FRP), carbon-fiber-reinforced polymer (CFRP), and glass-fiber-reinforced polymer (GFRP) as shear connectors to increase the thermal performance above that of the solid concrete rib while increasing the structural performance of ICSWP. Einea *et al.* [4] used the FRP shear connector to increase the thermal performance of ICSWP; the structural performance of the FRP was suitable for a shear connector. Bush and Stine [5] showed that the truss-shaped steel shear connector achieved a high degree of composite action, though they did not compare the steel shear connector to the other shear connector in terms of the degree of composite action and thermal performance. Salmon *et al*. [6] showed that the thermal performance of the truss-shaped FRP shear connector was higher than that of the truss-shaped steel shear connector, and the ultimate strength of the test specimens using the FRP was similar to the calculated value expected of full composite action. Frankl *et al*. [7,8] conducted a test using grid-shaped carbon-fiber-reinforced polymer (CFRP) as a shear connector to investigate the composite action. The degree of composite action of the test specimens was nearly 100% in terms of the initial stiffness in the experimental test; the degree of composite action of the test specimens in terms of the ultimate strength was not investigated. Morcous *et al*. [9] developed the NU-tie, which is similar to truss-shaped connector, to use the shear connector of the concrete sandwich panels and GFRP. The specimens with NU-tie achieved nearly full-composite action in terms of ultimate strength.

Previously studies on the configuration of shear connectors show that the continuous FRP shear connector can achieve a high degree of composite action and thermal performance. In addition, the bond between the insulation and concrete wythes affects the composite action. Woltman *et al*. [10] showed that the adhesion between the insulation and concrete has a significant initial effect on the strength of ICSWP initially that is not sustained under repeated loading. Tomlinson *et al*. [11] investigated the effect on ultimate strength of adhesion and friction between the insulation and concrete. The adhesion and friction contributed about half of the ultimate strength, though this was highly variable. Hassan and Rizkalla [12] conducted an experiment to investigate the effect of composite action via insulation materials. The expanded polystyrene (EPS) showed a higher composite action than extruded polystyrene (XPS), which exhibited a higher shear flow capacity. Oh *et al*. [13] show that the surface of XPS foam affects bond strength; however, they did not investigate the relationship between shear connector and bond. Soriano and Rizkalla [14] show that specimens using EPS foam and surface-sandblasted XPS foam have a higher shear strength than those using normal-surface XPS foam. These studies, however, focus on positive external force (wind pressure) in ultimate state; it is uncertain that ICSWP would show identical experimental results against a negative external force (wind suction) in ultimate state.

**Figure 1:**
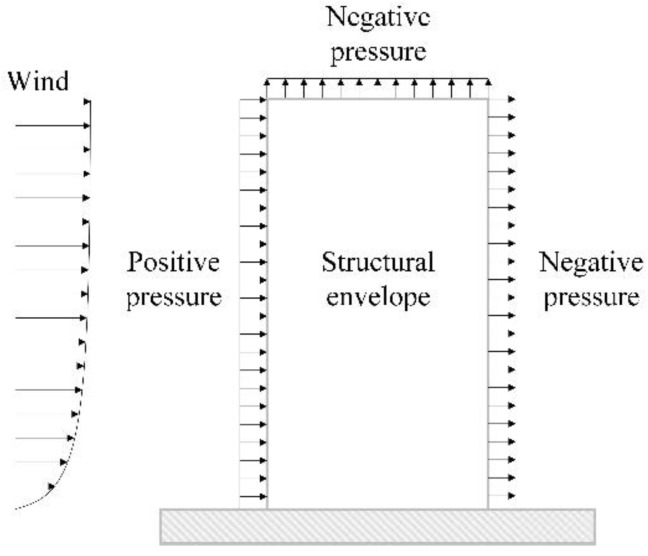
Effects of positive and negative pressure on the exterior wall of the building.

For ICSWP to be applied to high-rise buildings with strong wind loads, it should have sufficient resistance performance against frontal wind pressure (positive pressure) and back wind suction (negative pressure) (Figure 1). The main direction of wind loads acting on ICSWP is not fixed to one side; there is a possibility that it may be acting in both positive and negative directions. While the wind pressure, which is determined by the installed height of the ICSWP, has variable value according to the height, the wind suction, which is determined via the average height of the rooftop, has a constant value for one building. As the structural behavior of ICSWPs can be shown differently based on the direction of the wind load, it is necessary to compare and verify the structural behavior of ICSWPs under both positive and negative pressure.

In this study, the flexural behavior of ICSWPs is investigated through eighteen full-scale experimental tests. The positive and negative bending tests were configured to present wind pressure and suction. The degree of composite action was analyzed in terms of the surface roughness of the insulation, number of GFRP shear grids, and loading direction. The degree of composite action is computed via a simple analytical approach and compared to the test value in terms of the initial stiffness and ultimate strength. In addition, the failure mode of ICSWPs is investigated to analyze differences in the failure mechanism according to loading direction and the bond between concrete and insulation.

## 2. Materials and Experimental Test

### 2.1. Description of Test Specimen

**Figure 2:**
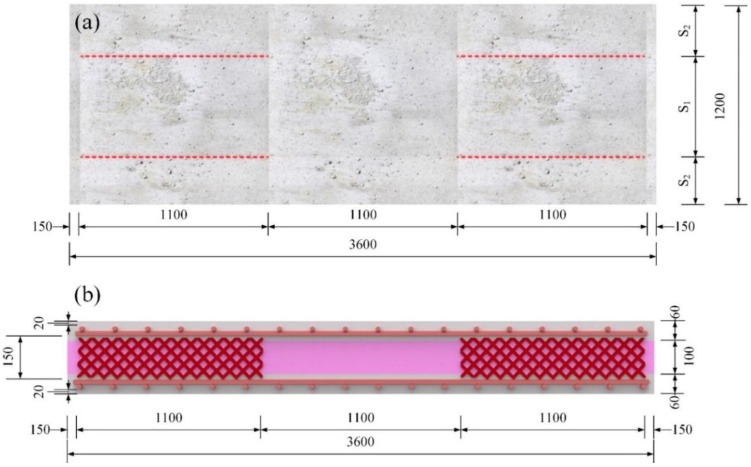
Typical dimension of specimens: (**a**) plan (2 grids on both shear spans); (**b**) section (2 grids on both shear spans).

**Figure 3:**
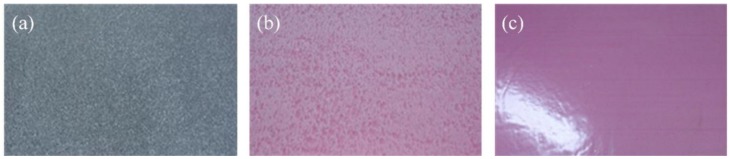
(**a**) Expanded polystyrene (EPS) foam; (**b**) extruded polystyrene (XPS) foam with roughened surface; (**c**) XPS foam with traction-free surface.

A total of eighteen full-scale specimens were tested to investigate the flexural behavior of ICSWPs. As shown in Figure 2, the typical shape and dimensions of the specimens were 1200 mm (b) × 3600 mm (l) × 220 mm (t). The thickness of each concrete wythe was 60 mm and insulation 100 mm in thickness was inserted between the concrete wythes. The types of insulations used are EPS, XPSST (XPS foam with roughened surface), where the surface was roughened, and XPSNB (XPS foam with traction-free surface), where the bond was removed by attaching a film on the surface of the XPS (Figure 3). Two to four GFRP shear grids with lengths of 1100 mm were inserted into the two wythes to enhance the composite behavior by transferring the shear flow between the two wythes shown in Figure 2a. The parameters S_1_ and S_2_ represent the interval distance between the GFRP shear grids and between the GFRP shear grids and the corner of the specimens, respectively, as shown in Table 1. Since the shear force was zero from a third point to two third point of the test specimen in four-point loading test, the GFRP shear grids did not install the part of the specimen (see reference [15] for detailed information).

To compare composite behavior according to the number of GFRP shear grids and the type of insulation in each load direction, the specimens were divided into three groups based on the type of insulation used, where each group used two to four GFRP shear grids (Table 1). A total of eighteen specimens were tested according to the load direction (positive or negative loads).

**Table 1:** Test specimens.

Load direction	No.	Label	Insulation type	No. of grids	S_1_ (mm)	S_2_ (mm)	GFRP strands
Positive	1	XPSST2_P	XPSST	2	300	600	4400TEX 3 strands
	2	XPSST3_P		3	200	400	
	3	XPSST4_P		4	150	300	
	4	XPSNB2_P	No bond	2	300	600	4400TEX 3 strands
	5	XPSNB3_P		3	200	400	
	6	XPSNB4_P		4	150	300	
	7	EPS2_P	EPS	2	300	600	4400TEX 3 strands
	8	EPS3_P		3	200	400	
	9	EPS4_P		4	150	300	
Negative	10	XPSST2_N	XPSST	2	300	600	4400TEX 3 strands
	11	XPSST3_N		3	200	400	
	12	XPSST4_N		4	150	300	
	13	XPSNB2_N	No bond	2	300	600	4400TEX 3 strands
	14	XPSNB3_N		3	200	400	
	15	XPSNB4_N		4	150	300	
	16	EPS2_N	EPS	2	300	600	4400TEX 3 strands
	17	EPS3_N		3	200	400	
	18	EPS4_N		4	150	300	

### 2.2. Material Properties

#### 2.2.1. Concrete

The design compressive strength of the concrete was 30 MPa, but the actual strength of the specimens was higher than the design strength. In the case of the positive loading test, on the test date, the concrete strength of XPSST_P specimens was 45 MPa, and that of the XPSNB_P and EPS_P specimens were 36 and 38 MPa, respectively. In the case of the negative loading test, on the test date, the concrete strength of the XPSST_N, XPSNB_N, and EPS_N specimens were 45, 44, and 45 MPa, respectively. Also, the slump test to check the concrete’s workability was conducted prior to the flexural test. In the case of the positive loading test, the slump of XPSST_P, XPSNB_P, and EPS_P groups are 179 mm, 184 mm, and 177 mm, respectively. In negative loading test, the slump of XPSST_N, XPSNB_N, and EPS_N groups are 183 mm, 178 mm, and 175 mm, respectively.

#### 2.2.2. Wire Mesh

Wire mesh with a diameter of 7 mm and spacing of 100 mm in both directions was used for flexural reinforcement of the concrete wythe. The reinforce ratio was 0.641%. The yield strength, tensile strength, and elastic modulus of the wire mesh are 534 MPa, 634 MPa, and 210,000 MPa.

#### 2.2.3. GFRP Shear Grids

The GFRP shear grid was fabricated by varying the horizontal and vertical cross directions. Identical tensile performance at the right angle should be realized to apply the GFRP shear grid to the ICSWPs, as the shear connectors are under identical loads from both directions. For identical strand strengths in the horizontal and vertical directions, three strands of 4400TEX, whose unit tensile strength is 6.3 KN, were used. The minimum spacing of the grids was fabricated to 35 mm × 35 mm to acquire a shear flow capacity of approximately 57.9 kN/m.

### 2.3. Test Parameters

The main parameter of the experimental program is the loading direction; the bond determined by type of insulation and the number of GFRP shear grids were additional parameters. Because the structural behavior of ICSWPs with equal configuration was expected to differ depending on loading direction, the flexural behavior of the full-scale specimens with the same insulation and number of GFRP shear grids was tested. It is possible to compare the degree of composite action of specimens in the experimental group with the same load direction according to the number of GFRP shear grids and type of insulation. XPSST and XPSNB specimens were used to evaluate the effect of the mechanical bond between the concrete wythes and the insulation based on surface roughness. EPS specimens were used to access the effect of the adhesive bond between the concrete wythes and the insulation based on absorptiveness according to load direction.

### 2.4. Test Setup and Procedure

**Figure 4:**
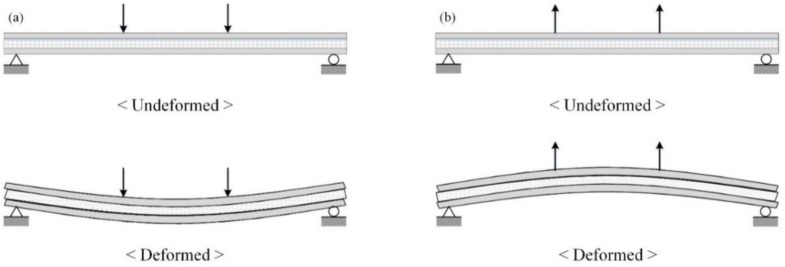
Diagram of the structural behavior of ICSWP specimens under (**a**) positive load (wind pressure) and (**b**) negative load (wind suction).

**Figure 5:**
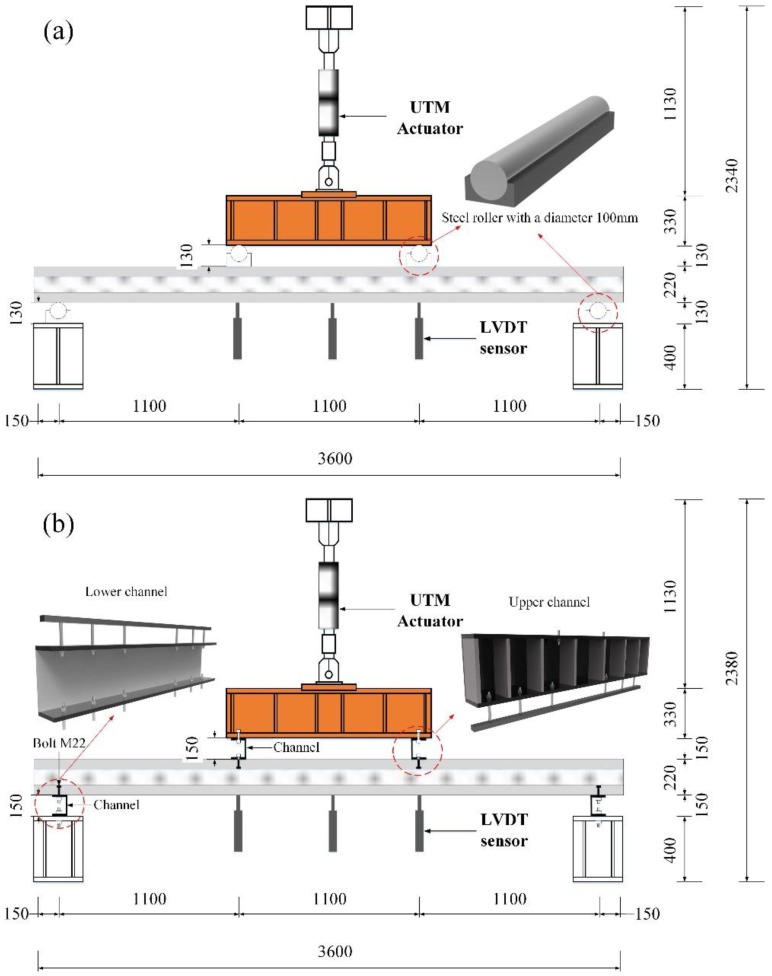
Experimental test-setup under (**a**) positive loading and (**b**) negative loading.

The Figure 4 presents a diagram of a four-point bending test of specimens under both positive and negative loads. The test setup is shown in Figure 5. A roller was used for both end supports and loading points in the positive loading test, but the tensile force of the negative loading test could not be implemented using the roller. Therefore, a negative load experimental condition was generated by connecting the channel to a pre-inserted bolt in the supports and loading points of the specimen.

The transfer beam reinforced with a stiffener was connected to a 2000 kN-capacity universal testing machine (UTM) to realize the positive and negative loading conditions. In the positive loading test, steel rollers with a diameter of 100 mm and length of 1200 mm were placed on two loading points and both end supports of the specimens. In negative loading test, the upper channel, connected with the reinforced beam, was reinforced with a stiffener to prevent deformation. The sectional properties of the upper channel with a length of 1200 mm are 110 mm (b) × 250 mm (h) × 15 mm (t_1_) × 9 mm (t_2_). The lower channel with a length of 1200 mm was installed to connect the specimens with both end supports; its sectional properties are 110 mm (b) × 300 mm (h) × 15 mm (t_1_) × 4 mm (t_2_). To connect the upper and lower channel with the specimen by the bolt, the steel bars were inserted in the specimen at both end supports and two loading points prior to concrete casting. The displacement-controlled loads were applied to conduct the flexural test, and all specimens were loaded at a rate of 0.05 mm/s to 25 mm and then at 0.1 mm/s to 100 mm. A total of six 200 mm LVDTs were installed at the central loading point and both loading points to compare and compensate for the displacement value at each position.

## 3. Results and Discussion

### 3.1. Load-Deflection Behavior Subjected to Positive and Negative Loading Tests

**Figure 6:**
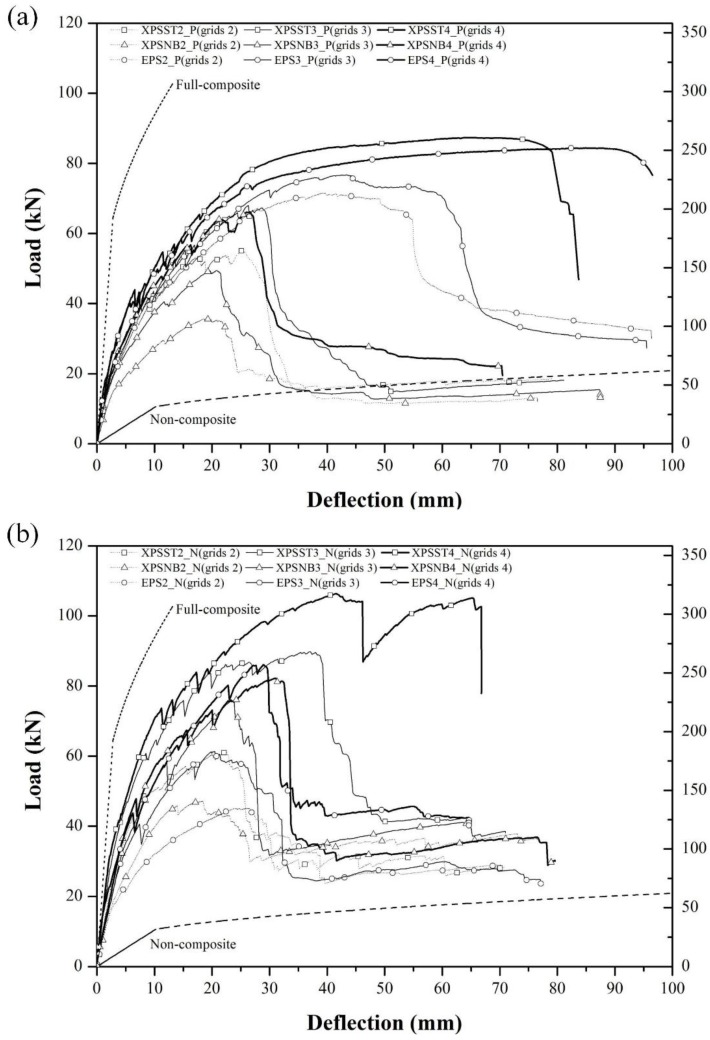
Load-deflection curve of specimens subjected to (**a**) positive load; and (**b**) negative load.

Figure 6 represents the load-deflection curves under positive and negative loading tests. Each load-deflection curve is analyzed according to the number of GFRP shear grids and the type of insulation to evaluate the ultimate strength and degree of composite action.

When examining the load-deflection curves under positive loads, the specimen behave elastically at the center deflection of 0–2 mm. A crack in the center of the lower concrete wythe was initiated at the center deflection of 2.65–3.8 mm. As a result, the slope of the load-deflection curve gradually decreased. The crack propagated continuously until the specimen failed and the GFRP shear grids progressively ruptured. All specimens except XPSST4_P and EPS4_P showed partial composite behavior in terms of the ultimate strength until all GFRP shear grids were ruptured. After the specimens had reached the maximum load of 35.6–75.8 kN, all GFRP grids ruptured, showing the non-composite behavior and resulting in a rapid reduction in the strength of the specimens. The GFRP grids in the XPSST4_P and EPS4_P specimens remained intact, reaching a maximum load of 87.4 and 84.4 kN at center deflections of 64.2 and 84.4 mm, respectively. After the maximum load was reached, XPSST4_P and EPS4_P specimens maintained composite behavior until the steel fracture in the center of the lower concrete wythe occurred at center deflections of 79.1 and 94.6 mm, respectively.

The ultimate strength, which exhibits an increasing trend in proportion to the number of GFRP grids, is the highest in the EPS_P group, followed by the XPSST_P and XPSNB_P groups. The ultimate strengths of the XPSST2_P, XPSST3_P, and XPSST4_P specimens are 55.0 kN, 68.0 kN, and 87.4 kN, respectively. The ultimate strengths of the XPSNB2_P, XPSNB3_P, and XPSNB4_P specimens are 35.6 kN, 49.5 kN, and 66.1 kN, respectively. The ultimate strengths of the EPS2_P, EPS3_P, and EPS4_P specimens are 71.6 kN, 76.8 kN, and 84.4 kN, respectively. As shown, the ultimate strengths of the XPSST_P and XPSNB_P groups are strongly dependent on the number of GFRP grids; this is not the case for the EPS_P group.

When examining the load-deflection curves under negative loads, the specimen can be seen to behave elastically at center deflection of 0–2 mm. A crack was initiated at the center of the lower concrete wythe at a center deflection of 2.57–3.48 mm, similar to the positive loading test. The specimens behaved in a manner similar to that in the positive loading test in terms of crack propagation and the progressive rupture of GFRP shear grids. All GFRP grids were ruptured after reaching the maximum load of 44.9–89.3 kN in all negative test specimens except the XPSST4_N. These specimens exhibited non-composite behavior, showing a rapid reduction in ultimate strength. The XPSST4_N specimen, whose steel fracture occurred only in the negative loading test, maintained composite behavior after reaching the maximum load of 105.9 kN at a center deflection of 39.6 mm, until an additional center deflection of 27.2 mm occurred. The EPS_P and EPS_N groups exhibited distinctly different load-deflection curves between positive and negative loading tests. The EPS_P group exhibited composite behavior after reaching ultimate strength until the occurrence of an additional center deflection of 11.6–13.6 mm, while the EPS_N group, like the XPSNB_P and XPSNB_N specimens, exhibited a rapid reduction in ultimate strength upon reaching ultimate strength. All GFRP grids rupture early under negative loading tests, because the bond between the EPS foam and concrete wythe is weakened under negative loads in a manner similar to that of the EPS_N and XPSNB_N specimens, in which the bond between the XPS foam and concrete wythes was removed.

The ultimate strength in negative loading test exhibits an increasing trend in proportion to the number of GFRP grids, and is highest in the XPSST_N group, followed by the XPSNB_N and EPS_N groups. The ultimate strengths of XPSST2_N, XPSST3_N, and XPSST4_N are 60.3 kN, 89.3 kN, and 105.9 kN, respectively. The ultimate strengths of XPSNB2_N, XPSNB3_N, and XPSNB4_N are 50.0 kN, 75.4 kN, and 82.0 kN, respectively. The ultimate strengths of the EPS2_P, EPS3_P, and EPS4_P specimens are 44.9 kN, 60.9 kN, and 85.2 kN, respectively. As with positive loading tests, the XPSST_N and XPSNB_N groups show that ultimate strength is governed by the number of GFRP grids; similar phenomena are observed in the EPS_N specimens. Via test results, we confirm that the flexural behavior of the test specimens is affected by loading direction, and may result from differences in loading transfer mechanism between positive and negative loading. Therefore, the failure modes of specimens are investigated in Section 3.2 to determine the difference between positive and negative loading.

### 3.2. Failure Mode of Specimens

**Figure 7:**
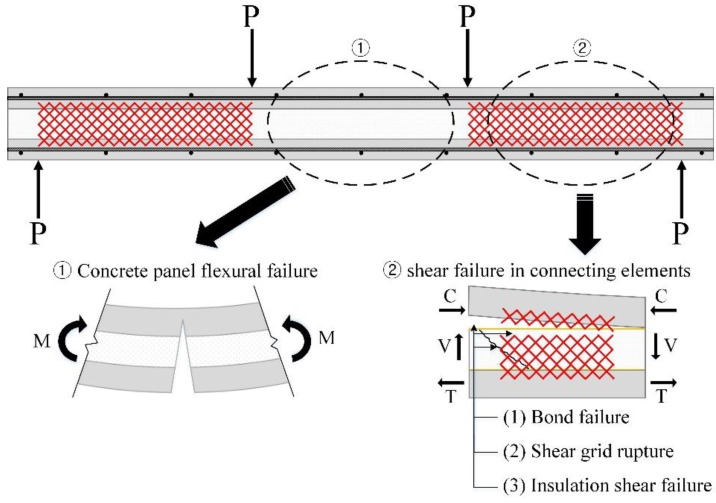
Flexural and shear failure mode of ICSWP specimens.

The inner/outer concrete wythes behaved as partial-composite members subjected to an external force. The bending moment, resulting from the external force acting on the two concrete wythes and shear flow resulting from the moment, will arise to the interface between the concrete wythes and insulation. If the connecting elements sufficiently withstand the shear flow, flexural failure of the concrete wythe will occur as the external force increases. If, due to the interaction of the connecting element, the shear strength does not have sufficient capacity for the shear flow, failure of the connecting element will occur prior to flexural failure of the concrete wythes. In other words, the failure mode of the specimens can be divided into two groups, as shown in Figure 7. The failure mode of each specimen is summarized in Table 2.

**Table 2:** The failure mode of each specimen according to load direction.

Label	Maximum Load (kN)	Deflection ^†^ (mm)	Failure Deflection (mm)	Failure Mode
XPSST2_P	55.0	25.1	26.0	Bond failure, shear grid rupture
XPSST3_P	68.0	26.3	29.4	Bond failure, shear grid rupture
XPSST4_P	87.4	64.2	79.1	Steel fracture
XPSST2_N	60.3	19.6	20.2	Bond failure, shear grid rupture
XPSST3_N	89.3	35.8	36.6	Bond failure, shear grid rupture
XPSST4_N	105.9	39.6	66.8	Steel fracture
XPSNB2_P	35.6	18.3	21.2	Bond failure, shear grid rupture
XPSNB3_P	49.5	19.5	21.3	Bond failure, shear grid rupture
XPSNB4_P	66.1	26.3	26.6	Bond failure, shear grid rupture
XPSNB2_N	50.0	17.8	18.8	Bond failure, shear grid rupture
XPSNB3_N	75.4	22.6	23.4	Bond failure, shear grid rupture
XPSNB4_N	82.0	30.5	31.9	Bond failure, shear grid rupture
EPS2_P	71.6	39.2	52.8	Insulation shear failure, shear grid rupture
EPS3_P	76.8	43.5	55.1	Insulation shear failure, shear grid rupture
EPS4_P	84.4	55.5	94.6	Steel fracture
EPS2_N	44.9	23.3	24.1	Bond failure, shear grid rupture
EPS3_N	60.9	19.0	19.9	Bond failure, shear grid rupture
EPS4_N	85.2	25.5	27.5	Bond failure, shear grid rupture

^†^ The deflection at the maximum load. Deflection differs from failure deflection, which is deflection at the failure mode.

**Figure 8:**
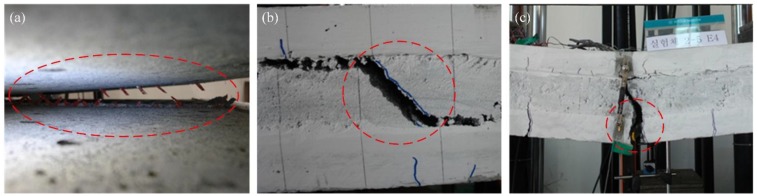
(**a**) Connecting element (bond, shear grid) failure of specimens with two or three GRFP shear grids; (**b**) shear failure of EPS foam in the positive loading test; (**c**) steel fracture of the concrete wythe in the EPS4_P specimen.

Because the shear strength of connecting elements for specimens with two or three GFRP shear grids is relatively lower than full-composite flexural strength (see Table 3 and Figure 12), the failure of the specimens occurred in the connecting elements (see Figure 8b). The steel fracture of the specimens with four GFRP shear grids occurred prior to the rupture of all GFRP shear grids due to the high shear capacity of the connecting elements (see Figure 8c); however, the failure mode of XPSNB4_P, XPSNB4_N, and EPS4_N, in which the shear strength of the connecting elements might be thought low from the results of the load-deflection curve, were bond failure and shear grid rupture.

The failure modes of the XPSST and XPSNB groups are the same regardless of loading direction, while different failure modes are observed between the EPS_P and EPS_N groups. The EPS_P group exhibited insulation shear failure rather than bond failure due to adhesive bonding, contributing to the high flexural strength of this group, though bond failure did occur prior to the insulation shear failure, because the adhesive bonds are easily broken through tensile force. The mechanical bond between the concrete wythes and XPS foam based on surface roughness are effective in both positive and negative loading tests. The maximum loads of the negative loading test of all except the EPS groups were generally higher than those of the positive loading test, indicating that the shear capacity of connecting elements increases under negative loading. The increment of maximum load is analyzed theoretically in Section 4.1, and the shear capacity of connecting elements is investigated in Section 4.2.

## 4. Composite Action

### 4.1. Degree of Composite Action

To analyze the degree of composite action, the theoretical degree of composite action of each specimen was calculated and analyzed in terms of the ultimate strength and initial stiffness. Equation (1) was used to calculate the theoretical load-deflection curve:

(1) $$\Delta =\frac{Pa}{24E_{c}I}\left(3L-4a^{2}\right)=\frac{23PL^{3}}{648E_{c}I}\;(\mathrm{for}\;a=L/3)$$

where Δ is the center deflection of the specimens; *P* is the acting load on specimens; L is the length of the specimens; E_c_ is the elastic modulus of concrete; and *I* is the moment of inertia.

The load-deflection curve was divided into two parts: before and after the concrete crack. The effective moment of inertia for cracked concrete, *I*_e_, was calculated using Equation (2) (see reference [16] Equations (8)–(9)):

(2) $$I_{e}={\left(\frac{M_{cr}}{M_{a}}\right)}^{3}I_{g}+\left[1-{\left(\frac{M_{cr}}{M_{a}}\right)}^{3}\right]I_{cr}$$

where *I*_g_ is the moment of inertia of the gross concrete section; *I*_cr_ is the moment of inertia of the cracked section transformed into concrete; *M*_a_ is the maximum moment in the member due to the service loads at the computed stage deflection, and *M*_cr_ is the cracking moment.

The method proposed by Benayoune *et al*., (2008) [17] and Mohamad *et al*., (2014) [18] was used to calculate the strength of the full- and non-composite actions at the ultimate stage. In the case of non-composite action, as each concrete wythe behaves independently, the ultimate moment capacity is determined by the sum of the moment capacity of each concrete wythe. In the case of full-composite action, as the two concrete wythes behave as one member, the inner/outer concrete wythes are considered one member, and the ultimate moment capacity is calculated from the product of the compressive force of the upper concrete wythe and the moment arm length between the center of the compressive and tensile forces of the reinforcement of the lower concrete wythe (see references [17] and [18] for further information). The theoretical calculated ultimate strengths of the full- and non-composite actions were 102.6 and 28.3 kN, respectively.

The degree of composite action for each specimen was evaluated in terms of the initial stiffness and ultimate strength. The method defined by Pessiki and MIynarczyk (2003) [2] was used for evaluation in terms of initial stiffness, where the degree of composite action was determined by comparing the experimental moment of the inertia of specimens (I_exp_) and the theoretical moment of inertia of full-composite action (I_c_) and non-composite action (I_nc_). Once the experimental moment of inertia of each specimen is calculated using Equation (4), Equation (3) is used to compare the degree of composite action of the specimens in terms of the initial stiffness (κ_1_). The degree of composite action in terms of the ultimate strength (κ_2_) is calculated using Equation (5). The degree of composite action based on the theoretical/experimental initial stiffness and ultimate strengths of the specimens is summarized in Table 3.

(3) $$\kappa_{1}=\frac{I_{\exp}-I_{nc}}{I_{c}-I_{nc}}(100)$$

(4) $$EI_{\exp}=\frac{23PL^{3}}{648\Delta }$$

(5) $$\kappa_{2}=\frac{P_{\exp}-P_{nc}}{P_{c}-P_{nc}}(100)$$

where *I*_exp_ is the experimentally-determined moment of inertia; *I*_c_ and *I*_nc_ are the theoretical, uncracked, full- and non-composite moments of inertia, respectively; *P*_exp_ is the experimental ultimate strength of specimens; and *P*_c_ and *P*_nc_ are the theoretical ultimate strengths of full- and non-composite action, respectively.

**Table 3:** Degree of composite action in terms of initial stiffness and ultimate strength.

Label	Degree of composite action in terms of initial stiffness				Degree of composite action in terms of ultimate strength	
	Cracking load (kN)	Displacement (mm)	I_exp_ (10^6^ mm^4^)	$\kappa_{1}$ (%)	Ultimate strength (kN)	$\kappa_{2}$ (%)
Full-composite	64.5	2.78	964.8	100	102.6	100
Non-composite	10.6	10.20	43.2	0	28.3	0
XPSST2_P	23.1	3.65	253.7	23	55.0	36
XPSST2_N	28.6	3.35	344.2	33	60.3	43
XPSST3_P	20.3	2.77	293.8	27	68.0	53
XPSST3_N	32.9	2.90	457.3	45	89.3	82
XPSST4_P	26.1	2.71	386.8	37	87.4	80
XPSST4_N	35.3	2.97	510.0	51	105.9	104
XPSNB2_P	15.8	3.25	218.9	19	35.6	10
XPSNB2_N	24.2	4.34	226.3	20	50.0	29
XPSNB3_P	23.4	4.03	260.8	24	49.5	29
XPSNB3_N	34.7	4.85	290.0	27	75.4	63
XPSNB4_P	25.9	3.53	330.3	31	66.1	51
XPSNB4_N	29.5	3.48	343.8	33	82.0	72
EPS2_P	20.8	3.33	274.5	25	71.6	58
EPS2_N	22.4	4.79	188.8	16	44.9	22
EPS3_P	26.5	3.77	306.9	29	76.8	65
EPS3_N	38.8	6.77	231.1	20	60.9	44
EPS4_P	26.4	2.76	417.8	41	84.4	76
EPS4_N	43.8	6.23	283.9	26	85.2	77

**Figure 9:**
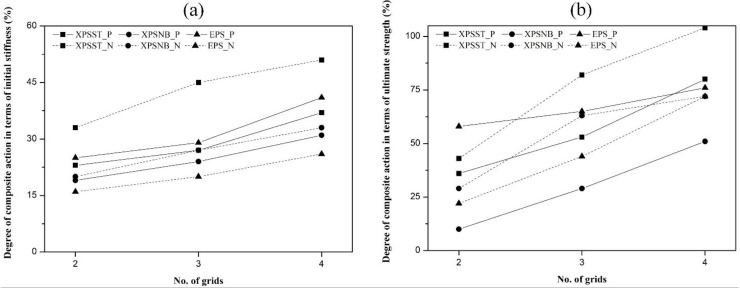
Degree of composite action, (**a**) initial stiffness and (**b**) ultimate strength.

In Table 3 and Figure 9, XPSST4_N specimens accomplished the highest degree of composite action, with a value of approximately 100% in terms of ultimate strength and 50% in terms of initial stiffness. The XPSNB2_P specimens showed the lowest degree of composite action, with a value of 10% in terms of ultimate strength and 19% in terms of initial stiffness. In the positive loading test, the degree of composite action in terms of initial stiffness and ultimate strength was the highest in the EPS_P groups, followed by the XPSST_P and XPSNB_P groups. The degree of composite action in terms of initial stiffness of the XPSST_P, XPSNB_P, and EPS_P groups increased on average by 7%, 6%, and 8%, respectively, per GFRP grid, and the degree of composite action in terms of ultimate strength of the XPSST_P, XPSNB_P, and EPS_P groups increased on average by 22%, 21%, and 9%, respectively, per GFRP grid. In the negative loading test, the degree of composite action in terms of initial stiffness and ultimate strength was the highest with the XPSST_N specimens, followed by the XPSNB_N and EPS_N specimens. The degree of composite action in terms of initial stiffness of the XPSST_N, XPSNB_N, and EPS_N groups increased on average by 9%, 7%, and 5%, respectively, per GFRP grid, and the degree of composite action in terms of ultimate strength of the XPSST_N, XPSNB_N, and EPS_N groups increased on average by 31%, 22%, and 27%, respectively, per GFRP grid.

All groups except the EPS_P group show that the ultimate strength is strongly dependent on the number of GFRP grids; the EPS_P group shows similar ultimate strength to the other group because the adhesive bonds contribute more to the composite action than the number of GFRP grids. Also, the degree of composite action according to the surface roughness is compared to determine the effect of the mechanical bond. The degree of composite action in terms of initial stiffness and ultimate strength of the XPSST_P group is higher, at 3%–6% and 24%–29%, than that of the XPSNB_P group, and that of the XPSST_N group are higher, at 13%–18% and 14%–32%, than that of the XPSNB_P group. The mechanical bond based on surface roughness has an effect on the composite action and is effective in both positive and negative loading tests.

It has been experimentally proven that the shear capacity of connecting elements, which is thought to affect the composite action of test specimens, changed depending on not only the type of insulation and the number of GFRP shear grids, but also the loading direction. For example, a higher degree of composite action was obtained under the negative loading test compared to the degree of composite action of the XPSST and XPSNB groups with equal GFRP shear grids according to loading direction. The EPS specimens with equal GFRP shear grids; however, exhibited a higher degree of composite action under the positive loading test. In other words, the shear capacity of connecting elements increases under negative loading more than under positive loading tests for XPSST and XPSNB specimens; the opposite results are observed in EPS specimens. Because the adhesive bond had no effect during the negative loading test, the mechanical bond strength is investigated to explain the higher degree of composite action. Also, there was the different grid shear flow strength between positive and negative loading test that contributes to the ultimate strength of the test specimens.

### 4.2. Shear Flow Capacity of Connecting Elements

The upper and lower concrete wythes are connected by connecting elements comprised of GFRP grids, insulation, and bond. If the shear flow capacity of the connecting elements are calculated from the shear flow strength of the GFRP grids only, the results of the higher degree of composite action of XPSST specimens than XPSNB specimens will not be reflected in the overall shear flow capacity of the connecting elements. Therefore, the shear capacity of the connecting elements is determined via the shear flow strength of the GFRP grids and the bond between the concrete wythes and insulation.

**Figure 10:**
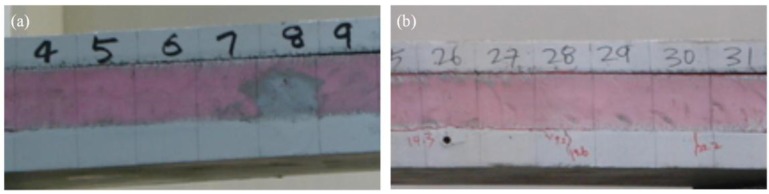
(**a**) XPSST2 slip at first load peak and (**b**) XPSNB3 slip at first load peak.

In this experimental test, the bonds can by divided into mechanical bonds based on friction between the XPSST foam and concrete wythe, and adhesive bonds based on the absorptiveness of the EPS foam. Adhesive bonds are difficult to quantify, while mechanical bonds can be determined experimentally through comparison of XPSST and XPSNB groups with equal numbers of GFRP grids. The first load peak, which was considered to be the bond strength of each specimen, is investigated in the load-deflection curve, and Figure 10 shows that the bond is partially removed at the first load peak. The value, with the bond strength of the XPSNB group subtracted from the bond strength of the XPSST group can be defined as the mechanical bond strength, as summarized in Table 4.

**Table 4:** Mechanical bond strength and effective shear flow strength according to load direction.

Load direction	No. of grids	First load peak (kN)		Mechanical bond strength (kN)	Ultimate strength of XPSST specimens (kN)	Effective shear flow strength (kN/m)
		XPSST	XPSNB			
Positive	2	40.4	20.7	19.7	55.0	52.8
	3	41.6	23.9	17.7	68.0	50.1
	4	45.8	26.4	19.4	87.4	50.7
Negative	2	48.5	25.6	22.9	60.3	55.8
	3	60.8	36.8	24	89.3	65.1
	4	65.8	47.9	17.9	105.9	65.7

**Figure 11:**
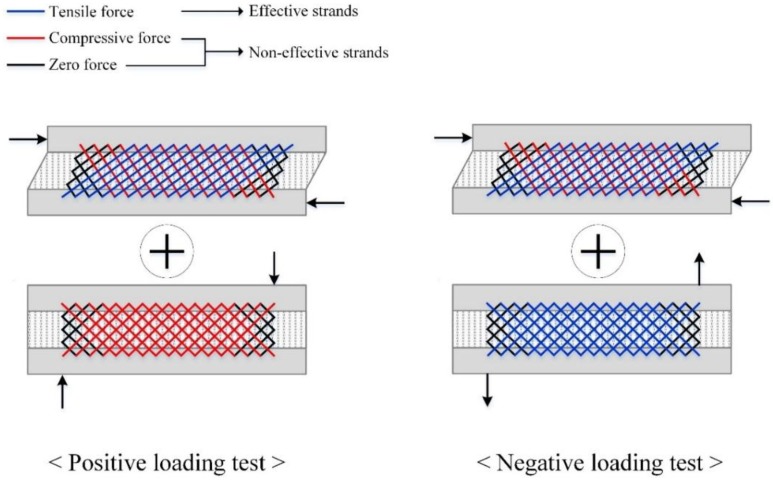
Conceptual effective strands of GFRP grids subjected to positive or negative loads.

The shear flow strength of the GFRP grids can be obtained theoretically from the number of effective strands subjected to tensile force, because GFRP grids are easily buckled when subjected to compressive force. Figure 11 represents the effective strands under positive and negative loading. Under positive loading, the tensile strength of the effective strands is weakened by external compressive force, possibly resulting in a lower experimental shear flow strength of a grid when compared to the theoretical shear flow strength, which is approximately 57.9 kN/m. A grid resists an external force of 19.4 kN with 13 effective strands (see reference [15] for further information). Additional strands may exist under negative loading, because the upper and lower concrete wythes received tensile force. In other words, the number of effective strands of a grid was not counted exactly under negative loading, but may have a higher value than under positive loading. If the mechanical bond strength obtained from each specimen is assumed to be valid until the GFRP grids fracture, the effective shear flow strength (q_c_) is defined in Equation (6) and the value summarized in Table 4. The term “n” in Equation (6) represents the number of GFRP grids. I and Q are the moment of inertia and the first moment of area, respectively, and the values of I and Q are 964,800,000 mm^4^, and 5,760,000 mm^3^, respectively (see reference [15] for a detailed calculation).

(6) $$q_{c}=\frac{\mathrm{Ultimate}\;\mathrm{strength}\;\mathrm{of}\;\mathrm{XPSST}-\mathrm{Mechanical}\;\mathrm{bond}\;\mathrm{strength}}{n}\times \frac{Q}{2I}$$

In Table 4, the effective shear flow strengths under positive and negative loading are on average 51.2 and 62.2 kN/m, respectively, and a grid of positive and negative loading test resist an external force of 17.1 and 20.8 kN, respectively. The effective shear flow strengths under negative loading are higher by 21% than under positive loading. The mechanical bond strengths under negative loading are higher by 2.7 kN than under positive loading due to the high clamping effect caused by the number of effective strands. The compressive force applied to concrete wythes results in a lower shear flow strength for the grid when compared to the theoretical value, as experimentally confirmed in the positive loading test. However, the tensile force applied to the concrete wythes creates a synergic effect on the shear flow strength of the grid, because it disturbs the buckling of the compressive strands and may increase the number of effective strands in the negative loading test.

**Figure 12:**
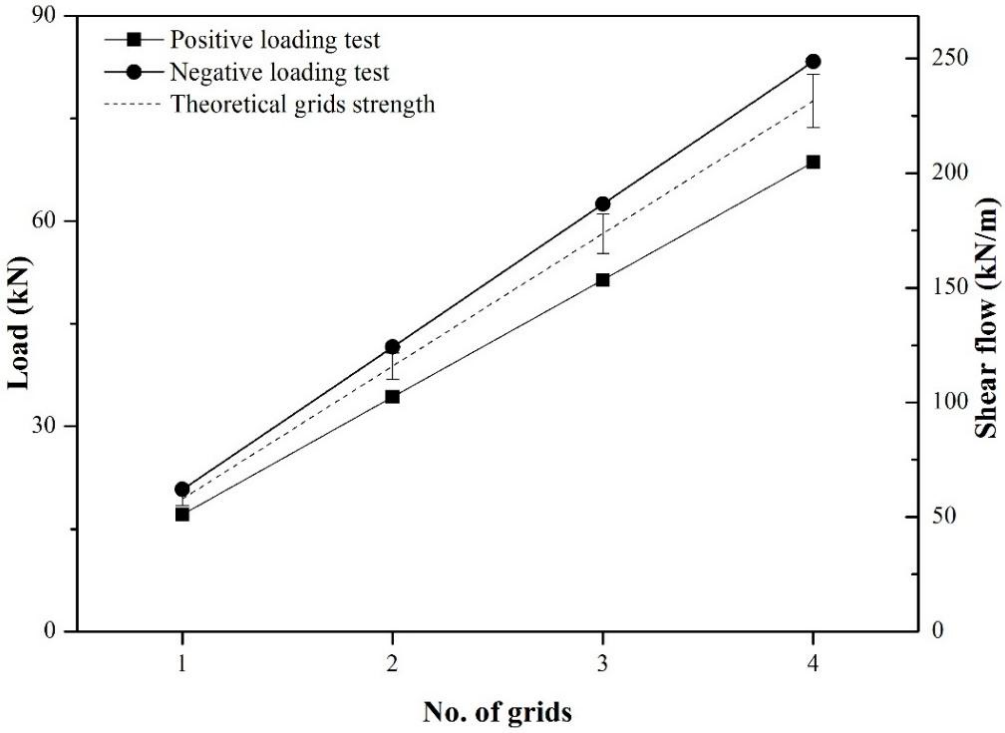
Experimental and theoretical effective shear flow strength.

Figure 12 represents a comparison between the experimental and theoretical shear flow strengths of GFRP grids under positive and negative loading. The experimental shear flow strengths under positive and negative loading are 88% and 107% of the theoretical value, respectively. This is the primary reason that the ultimate strength under negative loading is higher than under positive loading in the XPSST and XPSNB groups. An additional, minor contributor is an increase in the mechanical bond strength resulting from the clamping effect, which is determined via the number of effective strands. The ultimate strength of the EPS_N groups is lower than that of the EPS_P groups despite the increase in the shear flow strength of the GFRP grids, because the adhesive bond did not affect the flexural behavior under negative loading. Accordingly, the adhesive bond based on the absorptiveness of the EPS foam will not be considered in the design strength of ICSWPs, while the mechanical bond based on the surface roughness of the XPS foam may be considered due to its effect on ultimate strength under both positive and negative loading. Considering the lower tensile strength of effective strands under positive loading, the actual shear flow strength is approximately 0.85–0.90 times that of the theoretical shear flow strength. Additional tests will be conducted to investigate the mechanical bond strength according to the surface roughness of the XPS foam and the strength reduction factor of the GFRP grid in order to utilize the actual design.

## 5. Conclusions

In this study, the composite behavior of ICSWP reinforced with GFRP grids is investigated with respect to load direction, using both positive and negative loading. An experimental program, including eighteen full-scale specimens, was conducted with test variables, including the type of insulation, surface roughness, and number of GFRP shear grids. The failure modes of each specimen are examined to compare the structural behavior according to load direction. The degree of composite behavior is analyzed in terms of the initial stiffness, taking into account the varying moment of inertia due to crack propagation and ultimate strength. In addition, the mechanical bond strength based on surface roughness is analyzed at the first load peak and then effective shear flow strength of GFRP grids is calculated from the ultimate and mechanical bond strengths.

EPS_P group showed similar flexural strength with weak dependence on the number of shear grids. In this group, a material characteristic of absorptiveness in the EPS foam resulted in a high adhesive bond between the concrete and insulation that governed the flexural and composite behavior. However, the flexural strength of the EPS_N groups showed a dependence on the number of shear grids under negative loading, because the effect of the adhesive bonds were weakened by the tensile force. XPSST_P and XPSNB_P groups showed that flexural strength is governed by the number of shear grids; mechanical bonds based on surface roughness have a consistent effect on the initial stiffness and ultimate strength. Also, the flexural strength of the XPSST_N and XPSNB_N groups shows a tendency to depend on the number of shear grids, which is similar to the results of the positive loading test. We confirmed that the mechanical bond based on surface roughness is effective under negative as well as positive loading.

The higher degree of composite action under negative loading was obtained in XPSST and XPSNB groups. The primary reason for this was the difference in shear flow strength of each GFRP shear grid between positive and negative loading tests. In the case of negative loading, the tensile force acting on the shear grids from concrete wythe allowed the GFRP strands in both orthogonal directions to make some contribution to the load-carry capacity, and the effective strands resisted against the flexural-shear force. On the contrary, the compressive force acting on the shear grids from concrete wythes during positive loading weakened the load-carrying capacity of the shear grids. As result, the shear flow strength under negative loading was higher by approximately 21% than that under positive loading, contributing to the higher ultimate strength under negative loading.

The specimens in the EPS_N group showed that the adhesive bond is weakened in negative loading tests; thus, composite behavior based on the adhesive bond is not expected. The design strength of the ICSWP with EPS foam may be governed by the shear flow strength of grids. On the other hand, since the mechanical bond of the XPS foam are based on the surface roughness, they were effective under positive and negative loading tests, so it concludes that the composite behavior from mechanical bonds can be applied to the design strength of the ICSWP with continuous GFRP shear connectors.
